# A Multimodal MRI-Based Model for Colorectal Liver Metastasis Prediction: Integrating Radiomics, Deep Learning, and Clinical Features with SHAP Interpretation

**DOI:** 10.3390/curroncol32080431

**Published:** 2025-07-30

**Authors:** Xin Yan, Furui Duan, Lu Chen, Runhong Wang, Kexin Li, Qiao Sun, Kuang Fu

**Affiliations:** 1 Department of Radiology, The Second Affiliated Hospital of Harbin Medical University, Harbin 150086, China; yanxin20010326@163.com (X.Y.);; 2 PET/CT Department, The Second Affiliated Hospital of Harbin Medical University, Harbin 150086, China; 3 Department of Radiology, Jiaozhou Branch of Shanghai East Hospital, Tongji University, Jiaozhou 266300, China

**Keywords:** magnetic resonance imaging, colorectal cancer, radiomics, deep learning, SHAP

## Abstract

**Purpose**: Predicting colorectal cancer liver metastasis (CRLM) is essential for prognostic assessment. This study aims to develop and validate an interpretable multimodal machine learning framework based on multiparametric MRI for predicting CRLM, and to enhance the clinical interpretability of the model through SHapley Additive exPlanations (SHAP) analysis and deep learning visualization. **Methods**: This multicenter retrospective study included 463 patients with pathologically confirmed colorectal cancer from two institutions, divided into training (n = 256), internal testing (n = 111), and external validation (n = 96) sets. Radiomics features were extracted from manually segmented regions on axial T2-weighted imaging (T2WI) and diffusion-weighted imaging (DWI). Deep learning features were obtained from a pretrained ResNet101 network using the same MRI inputs. A least absolute shrinkage and selection operator (LASSO) logistic regression classifier was developed for clinical, radiomics, deep learning, and combined models. Model performance was evaluated by AUC, sensitivity, specificity, and F1-score. SHAP was used to assess feature contributions, and Grad-CAM was applied to visualize deep feature attention. **Results**: The combined model integrating features across the three modalities achieved the highest performance across all datasets, with AUCs of 0.889 (training), 0.838 (internal test), and 0.822 (external validation), outperforming single-modality models. Decision curve analysis (DCA) revealed enhanced clinical net benefit from the integrated model, while calibration curves confirmed its good predictive consistency. SHAP analysis revealed that radiomic features related to T2WI texture (e.g., *LargeDependenceLowGrayLevelEmphasis*) and clinical biomarkers (e.g., CA19-9) were among the most predictive for CRLM. Grad-CAM visualizations confirmed that the deep learning model focused on tumor regions consistent with radiological interpretation. **Conclusions**: This study presents a robust and interpretable multiparametric MRI-based model for noninvasively predicting liver metastasis in colorectal cancer patients. By integrating handcrafted radiomics and deep learning features, and enhancing transparency through SHAP and Grad-CAM, the model provides both high predictive performance and clinically meaningful explanations. These findings highlight its potential value as a decision-support tool for individualized risk assessment and treatment planning in the management of colorectal cancer.

## Simple Summary

This study developed and validated an interpretable machine learning model using multiparametric MRI (T2WI and DWI) to predict colorectal cancer liver metastasis (CRLM). It analyzed data from 463 patients across multiple centers, divided into training, internal testing, and external validation groups. The model integrated three data types: handcrafted radiomics features, deep learning features from ResNet101, and clinical data. This combined approach achieved the best performance (AUCs: 0.889 training, 0.838 internal test, 0.822 external validation), outperforming single-modality models. SHAP analysis identified key predictive features like specific T2WI textures (e.g., *LargeDependenceLowGrayLevelEmphasis*) and the biomarker CA19-9. Grad-CAM visualizations confirmed the deep learning model focused on relevant tumor regions. This accurate and explainable model shows potential as a clinical tool for personalized CRLM risk assessment and treatment planning.

## 1. Introduction

Colorectal cancer (CRC) ranks among the most prevalent malignancies globally, accounting for a significant proportion of cancer-related incidence and mortality [1]. Colorectal cancer patients exhibit a nearly 50% incidence of liver metastasis (colorectal cancer liver metastasis, CRLM), the highest rate among distant metastatic sites. This high frequency stems from the liver’s unique dual role. As the systemic metabolic gatekeeper organ, it is continuously exposed to gut-derived pathogens and metabolites via the gut–liver axis. Simultaneously, the liver’s inherent immunosuppressive microenvironment provides critical conditions for forming the pre-metastatic niche, as per the “seed-soil” theory, ultimately facilitating colorectal cancer cell colonization [2]. Liver metastasis is a critical prognostic determinant for primary CRC patients, as untreated cases exhibit a median survival of merely 6 months [3]. Multiple clinical factors have been established as associated with colorectal cancer liver metastases, including elevated serum levels of carcinoembryonic antigen (CEA) and carbohydrate antigen 19-9 (CA199) [4]. The current diagnostic approaches for colorectal cancer liver metastases, namely contrast-enhanced computed tomography (CT) or liver-specific magnetic resonance imaging (MRI) contrast agents, Positron Emission Tomography-Computed Tomography (PET-CT), and pathological liver biopsy, are associated with multiple limitations including invasiveness, safety concerns, and reliance on specialized expertise. Consequently, there remains a critical need to develop non-invasive predictive methodologies for CRLM that can be implemented through primary lesion MRI evaluation without requiring additional contrast agents.

MRI, particularly T2-weighted imaging (T2WI) and diffusion-weighted imaging (DWI) sequences, provides superior soft tissue resolution and critical insights into tumor microenvironments and water molecular diffusion, offering unique advantages in assessing tumor heterogeneity [5]. In recent years, radiomics—a non-invasive quantitative analysis technique—has gained prominence in oncology research by extracting subvisual features from conventional medical images. Through radiomics, key imaging biomarkers that are undetectable to the naked eye can be identified [6]. Studies demonstrate that radiomics-based models outperform traditional clinical models in CRC-related predictive tasks [7]. Concurrently, deep learning (DL), leveraging advanced artificial neural network architectures, has achieved remarkable progress in medical imaging, particularly in automated feature extraction and pattern recognition. Deep convolutional neural networks (CNNs) enable autonomous learning of high-order features, providing novel methodological frameworks for tumor evaluation and prediction [8,9,10]. Despite promising applications in various cancer studies, previous research has predominantly focused on single-modality radiomics or deep learning models. The integration of multimodal features for predicting CRLM remains largely unexplored.

Therefore, this study proposes a trimodal predictive model that integrates T2WI/DWI sequence-derived radiomic features, deep-learning features, and clinical biomarkers, distinguished by three key innovations: the elimination of direct liver scanning requirements; the ability to predict CRLM risk based solely on primary lesion MRI analysis; and the interpretable fusion of multimodal data through the Shapley Additive exPlanations (SHAP) method, which systematically quantifies the individual contribution of each input feature to model outputs [11,12]. The model aims to provide a non-invasive CRLM prediction tool for clinical practice, facilitating personalized decision-making and optimized screening protocols while ensuring transparency in feature attribution through SHAP-based interpretability.

## 2. Materials and Methods

### 2.1. Patients

The cohort comprised patients initially diagnosed with colorectal cancer at the radiology departments of two tertiary hospitals (The Second Affiliated Hospital of Harbin Medical University and Jiaozhou Branch of Shanghai East Hospital) in Northern China (designated as Center 1 and Center 2) between January 2020 and July 2024, with external validation conducted at Center 2. Inclusion criteria were (1) pathologically confirmed CRC diagnosis, patients with colorectal cancer liver metastases must have the presence of metastatic lesions confirmed by contrast-enhanced MRI of the liver or pathological biopsy of the liver; (2) no prior anticancer therapy before colorectal MRI examination; (3) availability of complete MRI datasets (T2WI and DWI); (4) complete clinical documentation. Exclusion criteria included (1) poor image quality with significant artifacts; (2) concurrent history of other malignancies. All baseline clinical characteristics were retrieved from medical records, including age, gender, tumor markers (CEA, normal range: 0.0–5.0 μg/L; CA19-9, normal range: 0.0–37.0 U/mL), MRI-reported T stage, and MRI-reported N stage. The T/N stages were evaluated on MRI by radiologist 1 (FK, 10 years of experience in abdominal imaging diagnosis) and radiologist 2 (ZM, 8 years of experience in abdominal imaging diagnosis), who were blinded to the clinical information. After consensus review by the two radiologists, 463 patients met the inclusion criteria and were ultimately enrolled in the study. The research protocol was approved by the Medical Ethics Committee of the Second Affiliated Hospital of Harbin Medical University accordance with the principles of Declaration of Helsinki. As the research was a retrospective study, the informed consents were waived off. The study adhered to the CheckList for EvaluAtion of Radiomics research (CLEAR) guidelines to ensure comprehensive and transparent reporting, and the details of the checklist are available in Table S1 [13].

We randomly divided the 367 CRC patients from Center 1 into training and internal test sets in a 7:3 ratio, while patients from Center 2 were used as an external validation set.

### 2.2. MRI Image Acquisition and Preprocessing

MRI examinations were performed using a GE Discovery MR750 3.0T scanner (General Electric Medical Systems Trade Development (Shanghai) Co., Ltd., Shanghai, China), with protocols including axial T1-weighted imaging (T1WI), axial T2-weighted imaging (T2WI), sagittal T2WI, coronal T2WI, fat-suppressed T2WI, and axial diffusion-weighted imaging (DWI). Key acquisition parameters were as follows: axial T2WI (echo time [TE] = 28 ms, repetition time [TR] = 3150 ms, slice thickness = 3 mm, matrix size = 256 × 256), and axial DWI (TE = 110 ms, TR = 6550 ms, slice thickness = 3 mm, field of view [FOV] = 300 mm × 300 mm, b-values = 0, 500, and 1000 s/mm^2^), ensuring standardized imaging across all participants. To minimize heterogeneity and scanner-related bias, all DICOM images were subjected to N4 bias field correction, normalized and resampled to the same resolution (1 mm × 1 mm × 1 mm).

### 2.3. Tumor Segmentation and Feature Extraction

Each region of interest (ROI) was created manually via the open-source ITK-SNAP (version 3.6.0, http://www.itksnap.org) by radiologist 1 and radiologist 2. A total of 1197 radiomic features per modality were extracted from segmented tumor volumes using PyRadiomics (version 3.1.0, https://pyradiomics.readthedocs.io/en/latest/index.html, accessed on 25 July 2024). This study used the pretrained CNN model, ResNet101, trained on the ImageNet dataset. The maximum cross-sectional area of each ROI was cropped and resized to 512 × 512 matrices to meet ResNet101 input specifications [14], with the architecture comprising an initial 7 × 7 convolutional layer, max-pooling layer, 33 bottleneck blocks (each containing 1 × 1, 3 × 3, and 1 × 1 convolutional layers), and a fully connected layer. Image patches encompassing > 75% tumor area were processed to derive 2048 deep-learning features from the penultimate layer. Gradient-weighted Class Activation Mapping (Grad-CAM) was employed to generate class-discriminative heatmaps using a jet color scheme (red: higher predictive relevance; blue: lower relevance), visualizing spatial attention patterns influencing CRLM predictions [15].

### 2.4. Feature Selection and Model Construction

**Figure 1:**
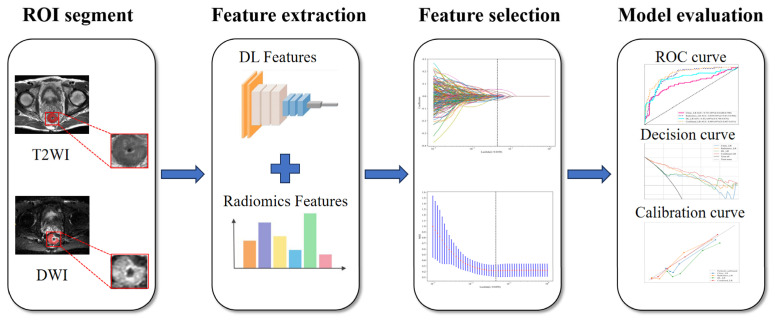
The workflow of this study. T2WI/DWI datasets were isotropically resampled and manually segmented by two radiologists. Radiomic features were extracted using PyRadiomics, while deep learning features were derived from tumor patches via ResNet101. Features with high inter-observer consistency were retained, standardized, and pruned to eliminate redundancy. Dimensionality reduction was performed using LASSO with cross-validation. Independent logistic regression models were constructed for radiomics, deep learning, and clinically significant predictors. Results analysis was performed through receiver operating characteristic (ROC) curves, decision curve analysis and calibration curves.

Initially, statistically significant clinical features were determined through univariate stepwise multivariate regression analysis performed on clinical variables in the training set. Concurrently, exploratory univariate and multivariate analyses were performed on the internal test set. Thereafter, to assess the reliability of radiomic features, inter-observer consistency was evaluated by comparing all features extracted from ROIs independently segmented by two radiologists, retaining those with intraclass correlation coefficients (ICC) ≥ 0.75. Features were then standardized via Z-score normalization using the mean and standard deviation derived from the training set to mitigate scale variability. For dimensionality reduction, Pearson correlation analysis was applied to quantify pairwise feature redundancy, with highly correlated features (r > 0.9) pruned to retain one representative variable, followed by Least Absolute Shrinkage and Selection Operator (LASSO) algorithm combined with 10-fold cross-validation to optimize feature selection while minimizing overfitting [16]. Conclusively, logistic regression (LR) models were independently constructed using filtered radiomic features (radiomics model), deep learning (DL) features (DL model), and clinical characteristics (clinical model) [17,18,19,20]. Finally, a combined model was developed by concatenating radiomic and DL features, reapplying LASSO for joint feature optimization, and incorporating clinical predictors to enhance CRLM discriminative capacity. A flowchart illustrating the study design can be seen in Figure 1.

### 2.5. Statistical Analysis

Statistical analyses and predictive modeling were conducted using R software (v4.2.1) and Python (v3.5.2). Continuous variables were compared via independent *t*-tests or Mann–Whitney U tests, while categorical variables were analyzed using chi-square or Fisher’s exact tests, with statistical significance defined as *p* < 0.05. Machine learning model development and evaluation were implemented through the Python “scikit-learn” package. Model performance was assessed using receiver operating characteristic (ROC) curves to calculate the area under the curve (AUC). To address class imbalance between CRLM-positive and CRLM-negative cases, F1-score and sensitivity were used as evaluation metrics to reduce bias from majority-class accuracy dominance. Accuracy, positive predictive value (PPV), and negative predictive value (NPV) were calculated to quantify the discrimination ability of the prediction models. Decision curve analysis (DCA) quantified clinical utility by estimating net benefit across threshold probabilities, while calibration curves evaluated agreement between predicted probabilities and observed outcomes. DeLong’s test was employed to compare AUC differences among models. Additionally, SHAP analysis elucidated feature contributions to predictions and explored associations between selected features and liver metastasis response.

## 3. Results

### 3.1. Patient Characteristics

A total of 463 CRC patients were enrolled in this study, comprising a training set (n = 256), an internal test set (n = 111), and an external validation set (n = 96), with the screening flowchart for Center 1 patients illustrated in Supplementary Figure S1. Liver metastases were identified in 79 (30.86%), 43 (38.74%), and 35 (36.46%) patients within the training, internal test, and validation sets, respectively. In accordance with randomized study design principles, formal testing for covariate imbalance was not performed, as any observed differences would inherently reflect random variation rather than systematic bias [21]. Comprehensive clinicopathological characteristics are summarized in Table 1.

### 3.2. Comparison Between Different Models

**Figure 2:**
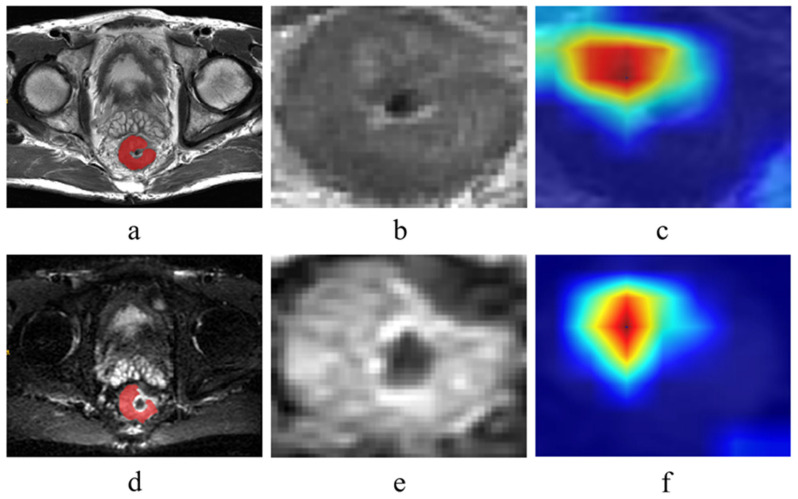
Two illustrative examples visualized using the Grad-CAM technique are presented, derived from MRI of the same patient at the identical anatomical slice level (maximum cross-sectional area). (**a**) T2WI region of interest (ROI), red indicates the manually delineated regions. (**b**) Maximum Cross-Section of ROIs from T2WI Sequences Employed in ResNet-101 Model Training. (**c**) T2WI heatmap. (**d**) DWI ROI, red indicates the manually delineated regions. (**e**) Maximum Cross-Section of ROIs from DWI Sequences Employed in ResNet-101 Model Training. (**f**) DWI heatmap. Red regions denote higher predictive values, while blue regions indicate lower predictive values in subfigures (**c**) and (**f**). The ResNet101 model predominantly activates regions within the tumor interior, demonstrating that the model correctly focuses on lesional areas for diagnostic predictions.

**Figure 3:**
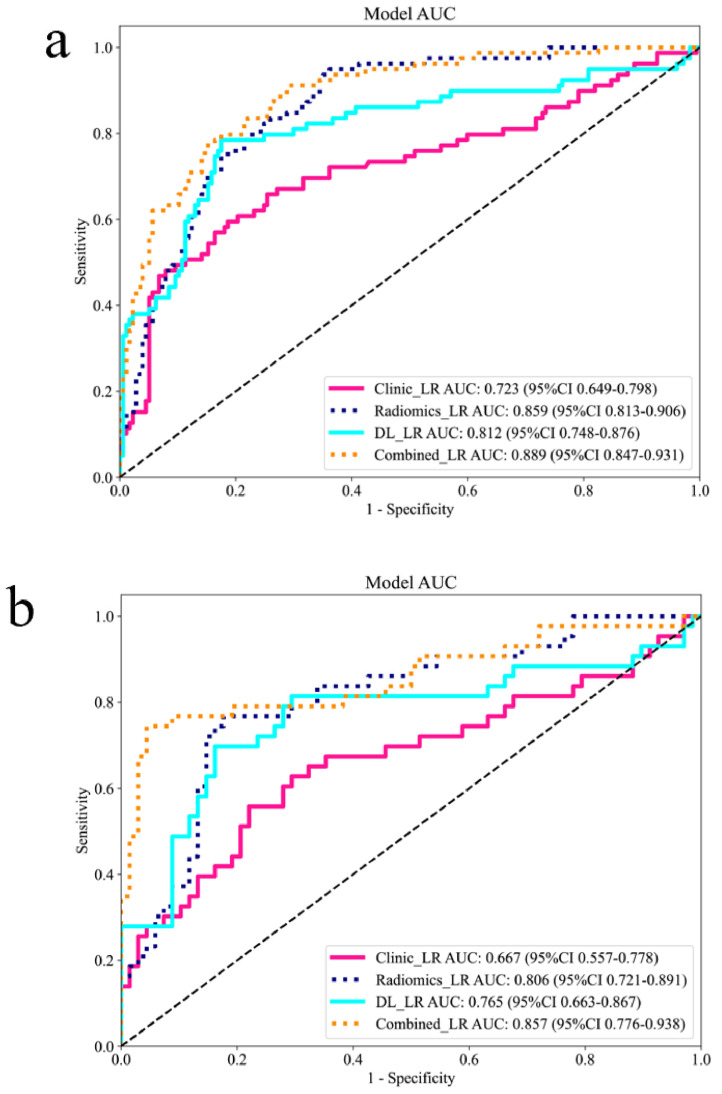

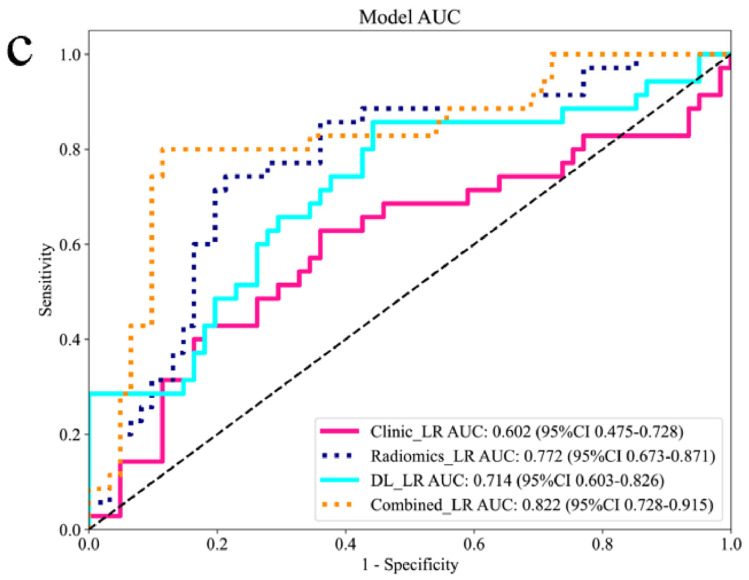
Performance of the four models. (**a**) ROC curve of the training set; (**b**) ROC curve of the test set; (**c**) ROC curve of the external validation set.

Figure 2 displays Grad-CAM heatmaps from the ResNet101 model, where color intensity reflects the relative importance of distinct MRI regions for predicting CRLM. The highest activation within tumor boundaries confirms the model’s accurate focus on lesional areas. Multivariable logistic regression analysis of the training set identified CA19-9 as the sole significant clinical predictor, while corresponding analysis for the internal test set is presented in Table 2. Figure 3 and Supplementary Figure S2 illustrates the performance of the clinical model (Clinic_LR), radiomics model (Radiomic_LR), deep learning model (DL_LR), and the combined model (Combined_LR), which integrates six radiomic features, four deep-learning features, and one clinical feature. Receiver operating characteristic (ROC) curves for the four models are shown in Figure 3a–c.

In the training set, the Combined_LR model achieved the highest AUC of 0.889 (95% CI: 0.847–0.931), with an accuracy of 0.793, sensitivity of 0.810, and specificity of 0.794. The DL_LR model yielded an AUC of 0.797 (95% CI: 0.738–0.856), the Radiomic_LR model an AUC of 0.859 (95% CI: 0.813–0.906), and the Clinic_LR model an AUC of 0.723 (95% CI: 0.649–0.798). In the internal test set, the Combined_LR model demonstrated an AUC of 0.838 (95% CI: 0.751–0.924). The DL_LR, Radiomic_LR, and Clinic_LR models achieved AUCs of 0.729 (95% CI: 0.630–0.828), 0.806 (95% CI: 0.721–0.891), and 0.667 (95% CI: 0.557–0.778), respectively. In the external validation set, the Combined_LR model attained an AUC of 0.822 (95% CI: 0.728–0.915). The DL_LR, Radiomic_LR, and Clinic_LR models showed AUCs of 0.714 (95% CI: 0.603–0.826), 0.772 (95% CI: 0.673–0.871), and 0.602 (95% CI: 0.475–0.728), respectively. DeLong’s test confirmed statistically significant differences in AUC values between the Combined_LR and unimodal models across all sets (*p* < 0.05). Decision curve analysis (DCA) revealed that the Combined_LR provided greater clinical net benefit compared to individual models, with detailed comparisons illustrated in Figure S2a–c. The calibration curves of the four models are shown in Supplementary Figure S2d–f. Table 3 and Supplementary Table S2 summarize the detailed performance comparison of the four models.

### 3.3. Interpreting the Model

**Figure 4:**
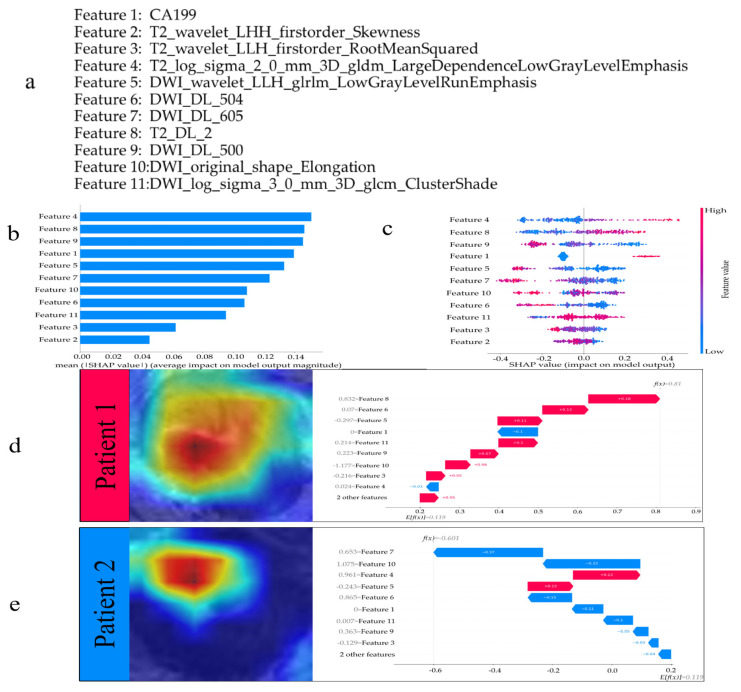
The model’s interpretation. (**a**) Features used for modeling. (**b**) The importance ranking of all variables according to the mean (|SHAP value|). (**c**) The importance ranking of all risk factors with stability and interpretation using the optimal model. The higher SHAP value of a feature is given, the higher risk of metastasis the patient would have. The red part in feature value represents higher value. The waterfall plot shows two examples of correct predictions, Patient 1 in (**d**) is an example of correctly predicting as positive, and Patient 2 in (**e**) is an example of correctly predicting as negative.

The SHAP method was employed to analyze the relationship between CRLM and the combined model, as illustrated in Figure 4. Figure 4a displays the features utilized for modeling. The interpretation of radiomic features is detailed in Supplementary Table S3. Figure 4b ranks feature importance based on mean absolute SHAP values. In Figure 4c, features are ordered by descending importance, with each sample represented by a colored dot. For instance, Feature 4 (*T2_log_sigma_2_0_mm_3D_gldm_LargeDependenceLowGrayLevelEmphasis*) quantifies the spatial aggregation of low-gray-level regions in T2WI, where blue dots indicate lower feature values associated with reduced likelihood of CRLM prediction (negative SHAP values on the horizontal axis), suggesting non-metastatic outcomes. Conversely, elevated values of *gldm_LargeDependenceLowGrayLevelEmphasis* may reflect increased low-gray-level clusters in T2WI, potentially indicative of tumor necrosis or heightened heterogeneity, and could correlate with CRLM progression. In the combined model, CA19-9, *T2_log_sigma_2_0_mm_3D_gldm_LargeDependenceLowGrayLevelEmphasis*, and *T2_DL_2* demonstrated positive correlations with CRLM, while *DWI_wavelet_LLH_glrlm_LowGrayLevelRunEmphasis* and *DWI_DL_500* exhibited negative correlations (Figure 4c). *T2_log_sigma_2_0_mm_3D_gldm_LargeDependenceLowGrayLevelEmphasis* emerged as the most critical feature (Figure 4c). To exemplify individualized interpretation, CRLM Patient 1 showed SHAP values exceeding the baseline, confirming metastatic status. Arrows denote feature contributions to the quantitative assessment (Figure 4d). Feature 8 (*T2_DL_2*) in this patient exhibited a value of 0.832, contributing positively to the SHAP value. In contrast, non-CRLM Patient 2 displayed SHAP values significantly below the baseline. Feature 7 (*DWI_DL_605*) with a value of 0.653 reduced the SHAP value (Figure 4e).

## 4. Discussion

Currently, there is no ideal method for the non-invasive detection of liver metastasis in colorectal cancer patients. This study established an interpretable predictive framework for liver metastasis detection in colorectal cancer by integrating multimodal magnetic resonance imaging (T2WI and DWI) with clinical biomarkers. The proposed multidimensional analysis paradigm synergistically combines conventional radiomic features, deep learning features, and clinical variables, demonstrating enhanced predictive accuracy compared with unimodal systems. The model achieved AUC values of 0.838 in the internal test set and 0.822 in the external validation set, confirming its robust generalizability across diverse patient populations. Our methodological innovation introduces SHAP analysis to interpret decision mechanisms, building on fused radiomics and deep learning features. This interpretable architecture not only identifies critical prognostic patterns but also provides clinically translatable evidence for metastasis risk stratification, addressing the crucial need for reliable prediction tools in precision oncology workflows.

Given the poor prognosis associated with liver metastasis in colorectal cancer (CRC) patients, research interest in prognostic biomarkers continues to grow. This study, through statistical analysis, identified CA19-9 as the biomarker with the greatest clinical value for predicting CRLM. Elevated CA19-9 levels correlate with more aggressive tumor biology. However, reliance solely on clinical factors demonstrates limited predictive power for CRLM (AUC: 0.602–0.723), highlighting an urgent need to develop more reliable predictive methods [22]. MRI serves as a cornerstone non-invasive imaging modality for TNM staging in colorectal cancer. However, current protocols for detecting hepatic metastases necessitate multiphase dynamic contrast-enhanced liver imaging, imposing substantial clinical and operational burdens. Traditional radiomics approaches in colorectal cancer research have been extensively validated through manual extraction of engineered features—including textural, morphological, and first-order statistical parameters—from MRI datasets. When integrated with statistical modeling and machine learning algorithms, these quantifiable biomarkers enable robust diagnostic classification, tumor grading, and prognostic evaluation. These methodologies demonstrate dual advantages: strong clinical interpretability through radiomic–physiological correlations and preservation of domain knowledge alignment throughout analytical workflows. For instance, Wang et al.’s study demonstrated that the performance of radiomics models incorporating clinical features was superior to that of single-feature models, further enhancing the predictive efficacy of the model [23]. The advent of deep learning, particularly convolutional neural networks (CNNs), has catalyzed a paradigm shift in medical image analysis [24]. Unlike traditional radiomics requiring manual feature engineering, CNNs autonomously extract hierarchical representations directly from raw imaging data, enabling end-to-end automated analysis pipelines. This data-driven approach eliminates multistep manual preprocessing inherent in conventional radiomics workflows. However, our experimental findings revealed a performance disparity where pure CNN architectures (e.g., ResNet101 pretrained on natural images) underperformed compared with radiomic feature-based models. This limitation likely stems from domain adaptation challenges between natural and medical imaging characteristics.

Furthermore, the inherent opacity of deep neural networks’ decision-making mechanisms poses critical clinical adoption barriers [25]. To address this translational gap, we implemented SHAP methodology, establishing mathematical interpretability frameworks that bridge learned computational biomarkers with pathophysiological correlates. SHAP employs game-theoretic principles to quantify feature contribution significance through Shapley value decomposition, generating human-interpretable attribution maps for complex model architectures. Within our multimodal integration framework, this method establishes comparative saliency maps that delineate the relative predictive weights among clinical parameters (e.g., serum CA19-9 levels), engineered radiomic features, and deep learning-derived biomarkers. Within the combined model, SHAP quantification identified T2WI-derived radiomic texture features as exhibiting maximal contribution weights. Higher SHAP values for this feature indicate an elevated probability of liver metastases predicted by the model. Elevated values of this feature corresponded to extensive low-gray-level dependencies in T2 images, potentially reflecting fibrotic/necrotic regions or malignant tumor textures that suggest aggressive metastatic potential. The radiomics features *gldm* and *glrlm* quantify spatial relationships between pixel pairs to characterize tumor heterogeneity, while deep learning features *T2_DL_2* and *DWI_DL_500* outperformed traditional texture features in predictive value. SHAP analysis demonstrated the superiority of multimodal models over single-feature approaches through effective feature weighting and cross-modal interaction, showing enhanced predictive accuracy with clinical applicability.

In external validation, the Combined_LR model demonstrated superior performance, achieving a positive predictive value (PPV) of 73.3% and a negative predictive value (NPV) of 85.2%. This indicates that approximately 73 out of every 100 patients predicted to have liver metastases were correctly identified, while approximately 85 out of every 100 patients predicted to be metastasis-free were truly negative. This statistically significant improvement over single-modality models (PPV < 66%) underscores the clinical value of multimodal integration in reducing false positives. Based on these robust predictive capabilities, we propose the following optimized clinical pathways: patients predicted positive (PPV ≥ 73%) should be directly referred to hepatobiliary surgery for assessment of resection or systemic therapy following diagnostic confirmation, while patients predicted negative (NPV ≥ 85%) may undergo routine 12-month surveillance without additional invasive investigations [26].

Our study has several limitations. For example, although this is a multicenter study, it only includes patients from two hospitals in Heilongjiang Province and Shandong Province of China, and the validity and universality of the research results need further verification. In the future, it will be necessary to build forecasting models based on a larger sample size. In addition, the proportion of positive cases in the sample is slightly unbalanced, which may lead to certain biases in the model output. Future work will involve collecting confirmed positive cases to enhance the predictive performance of our model. Our ultimate objective involves integrating this model into Picture Archiving and Communication Systems (PACS), thereby establishing a zero-interference clinical workflow from medical imaging data to diagnostic assistance.

## 5. Conclusions

This study verified that the model combining clinical, radiomics, and deep learning features is an effective method for predicting liver metastasis of colorectal cancer, and its effect is superior to that of models with single features. Moreover, we performed quantitative SHAP analysis to investigate how multimodal features influence model decisions. This approach clarifies the model’s decision-making process while providing a non-invasive method for predicting liver metastasis in colorectal cancer patients. Consequently, it enables personalized treatment planning.

## Figures and Tables

**Table 1:** Clinical Data of Sets.

Characteristics	Training Set		Internal Test Set		External Validation Set	
	Metastatic Group n = 79	Non-Metastatic Group n = 177	Metastatic Group n = 43	Non-Metastatic Group n = 68	Metastatic Group n = 35	Non-Metastatic Group n = 61
Age (mean ± SD)	60.85 ± 9.92	63.80 ± 11.22	61.70 ± 12.06	63.12 ± 11.31	62.55 ± 10.52	61.63 ± 10.87
Gender						
Female	19 (24.05%)	52 (29.38%)	17 (39.53%)	27 (39.71%)	15 (42.86%)	30 (49.18%)
Male	60 (75.95%)	125 (70.62%)	26 (60.47%)	41 (60.29%)	20 (57.14%)	31 (50.82%)
CEA, ng/mL						
≤5	34 (43.04%)	98 (55.37%)	17 (39.53%)	33 (48.53%)	18 (51.43%)	25 (40.98%)
>5	45 (56.96%)	79 (44.63%)	26 (60.47%)	35 (51.47%)	17 (48.57%)	36 (59.02%)
CA19-9, U/mL						
≤37	49 (62.03%)	159 (89.83%)	30 (69.77%)	56 (82.35%)	12 (34.29%)	18 (29.51%)
>37	30 (37.97%)	18 (10.17%)	13 (30.23%)	12 (17.65%)	23 (65.71%)	43 (70.49%)
T-stage						
T2	9 (11.39%)	49 (27.68%)	8 (18.60%)	13 (19.12%)	4 (11.43%)	16 (26.23%)
T3	52 (65.82%)	103 (58.19%)	25 (58.14%)	46 (67.65%)	20 (57.14%)	37 (60.66%)
T4	18 (16.80%)	25 (14.12%)	10 (23.26%)	9 (13.24%)	11 (31.43%)	8 (13.11%)
N-stage						
N0	9 (11.39%)	21 (11.86%)	4 (9.30%)	7 (10.29%)	6 (17.14%)	4 (6.56%)
N1	27 (34.18%)	56 (31.64%)	12 (27.91%)	19 (27.94%)	16 (45.71%)	39 (63.93%)
N2	43 (54.43%)	100 (56.50%)	27 (62.79%)	42 (61.76%)	13 (37.14%)	18 (29.51%)

SD, Standard Deviation; CEA, Carcinoembryonic Antigen; CA19-9, Carbohydrate Antigen 19-9.

**Table 2:** Results of Logistic Regression Analysis for Clinical Characteristics in the Internal test Set.

Characteristics	Univariate Analyses		Multivariate Analyses	
	OR (95% CI)	*p*-Value	OR (95% CI)	*p*-Value
Age	0.72 (0.86~0.99)	0.032	0.88 (0.77~1.00)	0.094
Gender (male)	1.25 (0.71~1.82)	0.491		
CEA (>5)	1.86 (1.03~2.48)	0.047	1.46 (0.80~1.59)	0.539
CA19-9 (>37)	3.58 (2.29~6.65)	<0.001	3.28 (1.64~6.01)	<0.001
T-stage	1.72 (1.20~2.48)	0.012	1.61 (0.92~2.59)	0.183
N-stage	1.00 (0.73~1.38)	0.964		

This analysis did not guide feature selection. Data in parentheses are reference level; Represents *p* < 0.05; OR, odds ratio.

**Table 3:** AUC Comparison of Prediction Models for Colorectal Cancer Liver Metastasis.

Models	AUC (95% CI)
Training Set	
Combined	0.889 (95% CI: 0.847–0.931)
DL	0.797 (95% CI: 0.738–0.856)
Radiomics	0.859 (95% CI: 0.813–0.906)
Clinic	0.723 (95% CI: 0.649–0.798)
Internal Test Set	
Combined	0.838 (95% CI: 0.751–0.924)
DL	0.729 (95% CI: 0.630–0.828)
Radiomics	0.806 (95% CI: 0.721–0.891)
Clinic	0.667 (95% CI: 0.557–0.778)
External validation set	
Combined	0.822 (95% CI: 0.728–0.915)
DL	0.714 (95% CI: 0.603–0.826)
Radiomics	0.772 (95% CI: 0.673–0.871)
Clinic	0.602 (95% CI: 0.475–0.728)

AUC, area under the receiver operating characteristic curve; CI, confidence interval.

## Data Availability

All data generated or analyzed during this study are available from the corresponding author on reasonable request.

## Supplementary Materials

The following supporting information can be downloaded at https://www.mdpi.com/article/10.3390/curroncol32080431/s1, Figure S1: Patient selection flowchart for Center 1; Figure S2: Performance of the four models; Table S1: CLEAR-S checklist (shortened version) without explanations; Table S2: Comparative Performance of Prediction Models for Colorectal Cancer Liver Metastasis; Table S3: Interpretation of Radiomics Features.

## Abbreviations

CRLM	Colorectal Cancer Liver Metastasis
SHAP	SHapley Additive exPlanations
T2WI	T2-weighted Imaging
DWI	Diffusion-weighted Imaging
LASSO	Least Absolute Shrinkage and Selection Operator
DCA	Decision Curve Analysis
CRC	Colorectal Cancer
CNNs	Convolutional Neural Networks
CEA	Carcinoembryonic Antigen
CA19-9	Carbohydrate Antigen 19-9
ROI	Region Of Interest
Grad-CAM	Gradient-weighted Class Activation Mapping
ICC	Intraclass Correlation Coefficients
LR	Logistic Regression
DL	Deep Learning
AUC	Area Under the Curve
