# Supplementary material for: A Multimodal MRI-Based Model for Colorectal Liver Metastasis Prediction: Integrating Radiomics, Deep Learning, and Clinical Features with SHAP Interpretation
Source: Curr Oncol. 2025 Jul 30;32(8):431. doi: 10.3390/curroncol32080431 (PMC12384400; doi:10.3390/curroncol32080431)
Supplement: Supplementary file 1 [file curroncol-32-00431-s001.zip › curroncol-3758569-supplementary.pdf]

Figure S1. Patient selection flowchart for Center 1.

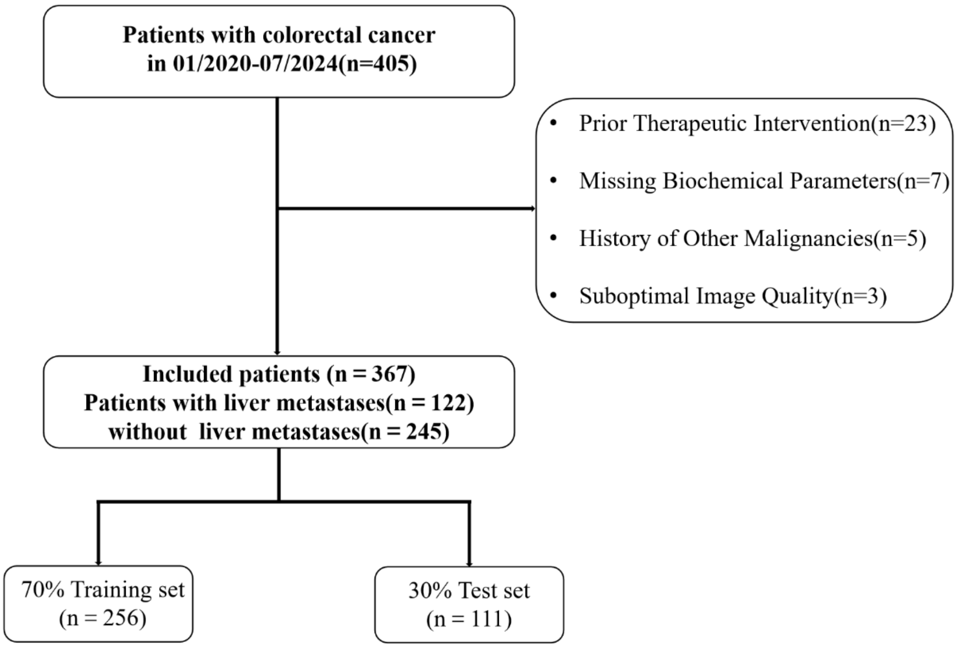

Figure S2. Performance of the four models. (a) DCA curve of the training set; (b) DCA curve of the test set; (c) DCA curve of the external validation set; DCA, decision curve analysis. (d) Calibration curves of the training set; (e) Calibration curves of the test set; (f) Calibration curves of the external validationset.

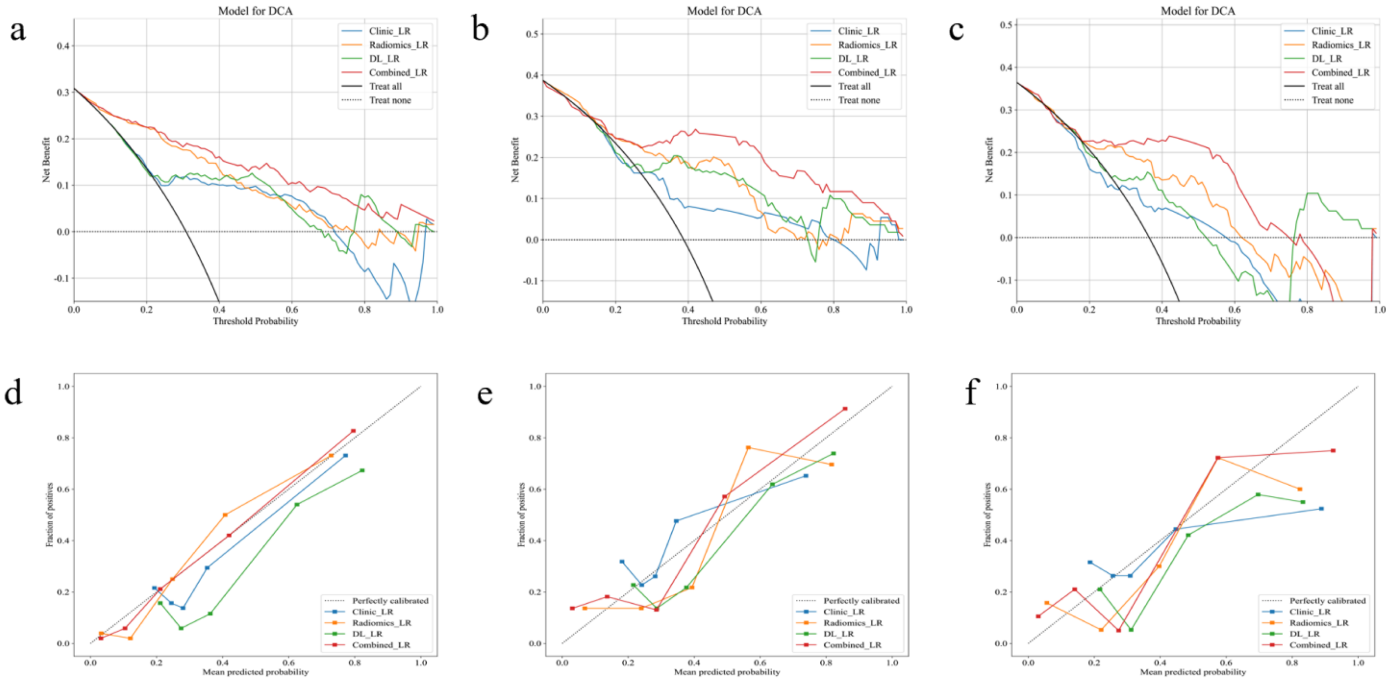

Table S1. CLEAR-S checklist (shortened version) without explanations.

Section	No.	Item	Yes	No	n/a	Page
**Method**						
Study Design	7	Adherence to guidelines or checklists (e.g., CLEAR checklist)	√	☐	☐	5
	8	Ethical details (approval)	√	☐	☐	5
	9	Sample size calculation	√	☐	☐	6
	10	Study nature (retrospective)	√	☐	☐	5
	11	Eligibility criteria	☐	☐	√	
	12	Flowchart for technical pipeline	√	☐	☐	8
Data	13	Data source (private)	√	☐	☐	5
	14	Data overlap	☐	√	☐	
	15	Data split methodology	√	☐	☐	6
	16	Imaging protocol (i.e., image acquisition and processing)	√	☐	☐	6
	17	Definition of non-radiomic predictor variables	☐	☐	√	
	18	Definition of the reference standard (i.e., outcome variable)	☐	☐	√	
Segmentation	19	Segmentation strategy	√	☐	☐	6
	20	Details of operators performing segmentation	☐	☐	√	
Pre-processing	21	Image pre-processing details	√	☐	☐	6
	22	Resampling method and its parameters	√	☐	☐	6
	23	Discretization method and its parameters	☐	☐	√	
	24	Image types (transformed)	√	☐	☐	6
Feature extraction	25	Feature extraction method	√	☐	☐	6
	26	Feature classes	√	☐	☐	7
	27	Number of features	√	☐	☐	6
	28	Default configuration statement for remaining parameters	☐	☐	√	
Data preparation	29	Handling of missing data	☐	√	☐	
	30	Details of class imbalance	√	☐	☐	8
	31	Details of segmentation reliability analysis	√	☐	☐	7
	32	Feature scaling details (e.g., normalization, standardization)	√	☐	☐	7
	33	Dimension reduction details	√	☐	☐	8
Modeling	34	Algorithm details	☐	√	☐	
	35	Training and tuning details	☐	√	☐	
	36	Handling of confounders	☐	☐	√	
	37	Model selection strategy	√	☐	☐	8
Evaluation	38	Testing technique (e.g., internal, external)	√	☐	☐	9
	39	Performance metrics and rationale for choosing	√	☐	☐	10
	40	Uncertainty evaluation and measures (e.g., confidence intervals)	√	☐	☐	10
	41	Statistical performance comparison (e.g., DeLong's test)	√	☐	☐	11
	42	Comparison with non-radiomic and combined methods	√	☐	☐	11
	43	Interpretability and explainability methods	√	☐	☐	11
**Open Science**						
Data availability	53	Sharing images along with segmentation data [n/e]	☐	√	☐	
	54	Sharing radiomic feature data	☐	☐	√	
Code availability	55	Sharing pre-processing scripts or settings	☐	√	☐	
	56	Sharing source code for modeling	☐	☐	√	

Model availability	57	Sharing final model files	☐	☐	√	
	58	Sharing a ready-to-use system [n/e]	☐	☐	√	

Yes, details provided; **No**, details not provided; **n/e**, not essential; **n/a**, not applicable. Note: Use the checklist in conjunction with the main text for clarification of all items. Fill the "Page" column with the related page number where the information is provided.

Table S2. Comparative Performance of Prediction Models for Colorectal Cancer Liver Metastasis.

Models	Sensitivity	Specificity	Accuracy	F1-score	PPV	NPV
Training Set						
Combined	0.810	0.794	0.797	0.707	67.2%	89.2%
DL	0.707	0.742	0.730	0.618	52.9%	86.3%
Radiomics	0.743	0.707	0.719	0.619	54.8%	85.3%
Clinic	0.714	0.667	0.683	0.581	47.1%	84.6%
Internal Test Set						
Combined	0.764	0.779	0.775	0.724	70.6%	81.8%
DL	0.687	0.806	0.760	0.689	66.7%	82.4%
Radiomics	0.693	0.746	0.726	0.661	59.6%	81.3%
Clinic	0.720	0.622	0.660	0.621	50.0%	81.0%
External validation set						
Combined	0.795	0.803	0.799	0.742	73.3%	85.2%
DL	0.784	0.773	0.777	0.720	65.9%	86.2%
Radiomics	0.653	0.761	0.722	0.631	55.6%	82.1%
Clinic	0.702	0.649	0.668	0.607	48.5%	81.7%

Table S3. Interpretation of Radiomics Features.

T2_wavelet_LHH_firstorder_Skewness	This feature quantifies the presence of limited extremely hyperintense or hypointense pixels within the region of interest, indicating whether these outliers induce left-skewed or right-skewed asymmetry in the overall intensity distribution.
T2_wavelet_LLH_firstorder_RootMeanSquared	This metric characterizes the signal intensity magnitude (particularly high-intensity signals) and measures the "energy" or intensity variation along the Z-axis direction within the region.
T2_log_sigma_2_0_mm_3D_gldm_LargeDependenceLowGrayLevelEmphasis	It emphasizes the prevalence and significance of large contiguous regions characterized by markedly hypointense (low-gray-level) voxels exhibiting high internal homogeneity.
DWI_wavelet_LLH_glrlm_LowGrayLevelRunEmphasis	This texture descriptor evaluates the continuity and homogeneity of hypointense regions in DWI images at the specified wavelet decomposition scale (LLH).
DWI_original_shape_Elongation	As a fundamental 3D morphological characteristic, it specifically quantifies the degree of elongation of observed targets (e.g., tumors) in DWI images within planes perpendicular to their longest axis.

DWI_log_sigma_3_0_mm_3D_glcm_ClusterShade	This feature represents texture patterns exhibiting pronounced spatial heterogeneity in 3D signal intensity distribution, characterized by high-contrast bright/dark regions with asymmetrical spatial arrangements.
